# Factor VII Activating Protease (FSAP) and Its Importance in Hemostasis—Part I: FSAP Structure, Synthesis and Activity Regulation: A Narrative Review

**DOI:** 10.3390/ijms24065473

**Published:** 2023-03-13

**Authors:** Iga Kwiatkowska, Ewa Żekanowska, Simona Lattanzi, Andrea M. Alexandre, Agata Kister-Kowalska, Artur Słomka

**Affiliations:** 1 Department of Pathophysiology, Nicolaus Copernicus University in Toruń, Ludwik Rydygier Collegium Medicum, 85-094 Bydgoszcz, Poland; iga.kwiatkowska@cm.umk.pl (I.K.); zorba@cm.umk.pl (E.Ż.); 2 Neurological Clinic, Department of Experimental and Clinical Medicine, Marche Polytechnic University, 60121 Ancona, Italy; alfierelattanzisimona@gmail.com; 3 UOC Radiologia e Neuroradiologia, Dipartimento di Diagnostica per Immagini, Radioterapia Oncologica ed Ematologia, Fondazione Policlinico Universitario A. Gemelli IRCCS, 00168 Rome, Italy; andrea.alexandre@policlinicogemelli.it; 4 Department of Pathomorphology, Placentology and Clinical Hematopathology, Jan Biziel University Hospital No. 2, 85-168 Bydgoszcz, Poland; agata.kister-kowalska@biziel.pl

**Keywords:** factor VII activating protease, coagulation, hemostasis

## Abstract

Factor VII activating protease (FSAP) was first isolated from human plasma less than 30 years ago. Since then, many research groups have described the biological properties of this protease and its role in hemostasis and other processes in humans and other animals. With the progress of knowledge about the structure of FSAP, several of its relationships with other proteins or chemical compounds that may modulate its activity have been explained. These mutual axes are described in the present narrative review. The first part of our series of manuscripts on FSAP describes the structure of this protein and the processes leading to the enhancement and inhibition of its activities. The following parts, II and III, concern the role of FSAP in hemostasis and in the pathophysiology of human diseases, with particular emphasis on cardiovascular diseases.

## 1. Prima Facie of Factor VII Activating Protease (FSAP)

The first reference to factor VII activating protease (FSAP) appeared in the study by Choi-Miura et al., dated 1996 [1]. Researchers purified this protein from human plasma using the affinity chromatography on hyaluronan-conjugated Sepharose and named it plasma hyaluronan-binding protein (PHBP) [1]. In 1999, Hunfeld et al. described, during the purification of vitamin K-dependent coagulation factors from human plasma, a novel plasma hyaluronan-binding serine protease (PHBSP) that exhibited amidolytic activity [2]. The authors implied that PHBP and PHBSP are proteins that originate from the same precursor [1]. Presently, the most commonly used name for this protein is factor VII activating protease (FSAP), which was named as such in the study by Römisch et al., indicating its role in factor VII (FVII) activation irrespectively of tissue factor (TF) [3]. Taken together, there are several names for FSAP present in the literature, i.e., PHBP [4,5,6,7,8,9,10], PHBSP [11], and hyaluronic acid binding protein 2 (HABP2) [12,13,14]; however, the authors studied the same protein.

The FSAP molecule is expressed from the single copy *HABP2* gene (35 kilobases (kb) in length, 12 introns, 13 exons) localized on chromosome 10q25-q26 [4]. The *HABP2* gene shows similarity to the genes of other serine proteases, such as factor XII (FXII), tissue plasminogen activator (tPA), and urokinase plasminogen activator (uPA) [4]. The *PLAU* gene encoding uPA is located near the *HABP2* gene, on chromosome 10q24 [15]. Considering these observations, the hypothesis that the *HABP2* gene emerged from the *PLAU* gene seems reasonable [4]. The previously mentioned similarities between *HABP2* and other genes encoding hemostatic-related proteins encouraged further experiments focused on FSAP functions.

The detailed analysis of *HABP2* gene revealed the promoter sequence that is directly upstream of the transcription start site and occurs in humans [16] and mice [4,16]. The promoter sequence of *HABP2* contains binding sites for transcription factors, including activator protein 1 (AP-1), specificity protein 1 (SP-1), hepatocyte nuclear factor 1 alpha (HNF1α) [4,16], hepatocyte nuclear factor 3 beta (HNF3β), activating transcription factor 3 (ATF3), c-fos, and CCAAT/enhancer binding protein delta (C/EBPδ) [16]. These data might be helpful to understand the molecular basis of FSAP functionality.

### 1.1. Sites of FSAP Synthesis

FSAP is produced mainly by the liver, which was shown both in animal [17,18] and human studies [1,4,7,19]. Besides hepatic sources, FSAP messenger RNA (mRNA) has been found in the murine [7] and human kidney [1], human pancreas and skeletal muscle [1]. The authors of the cited manuscript [1] postulated that the human brain and heart could not synthesize FSAP. More specifically, a further study confirmed that neurons and astrocytes did not express FSAP [20]. Different observations demonstrating contradictory evidence of FSAP synthesis occurring in human lung and placenta tissue have been described [1,21,22,23,24]. Choi-Miura et al.’s pioneering work did not observe FSAP mRNA expression in the human lung and placenta [1]. Contrastingly, Mu et al. [25], Wygrecka et al. [21], and Knoblauch et al. [22,23] found the rat [25] and human [21,22,23] lung to express FSAP protein, although FSAP expression was limited to alveolar macrophages both in rats [25] and humans [21]. The expression degree varied between these mammals. In rats, FSAP signal in immunohistochemical staining was weak and shown by only a small number of alveolar macrophages [25]. In alveolar macrophages from human tissue, the protein staining for FSAP was strong, but FSAP mRNA level was very minor [21]. The series of experiments using a mouse cell line indicated that FSAP protein was internalized by alveolar macrophages and degraded in their lysosomes, casting doubt on the contribution of these cells to FSAP synthesis [21]. In the case of the human placenta, Parahuleva et al. demonstrated this organ to synthesize FSAP [24]. FSAP mRNA and protein in the placenta were shown to gradually decrease with gestational age [24]. This increased expression in first trimester placenta might be associated with the FSAP-driven stimulation of the human trophoblast migration [24].

**Figure 1:**
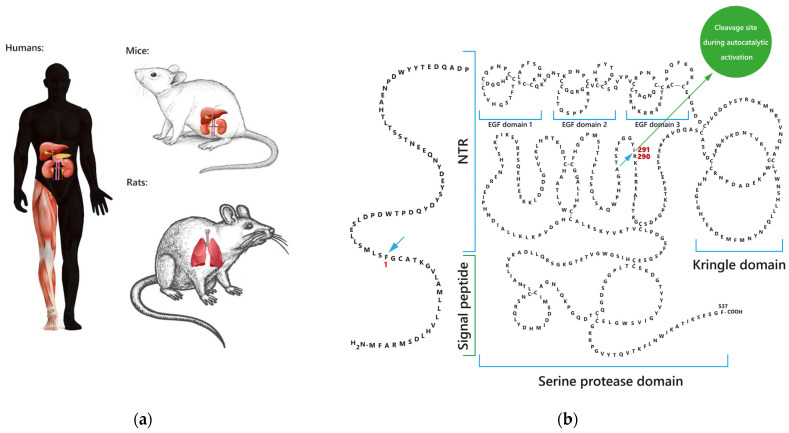
The places of factor VII activating protease (FSAP) synthesis and its structure: (**a**) FSAP is found in the liver, kidney, pancreas, and skeletal muscle in humans as well as in the liver and kidney in mice. A weak FSAP signal was observed in the lungs of rats; (**b**) regions of single-chain FSAP (scFSAP) structure are N-terminal region (NTR), three epidermal growth factor (EGF) domains (EGF1, EGF2, and EGF3), kringle domain, and the C-terminal serine protease domain. The signal peptide of FSAP contains 23 amino acids.

The ability of epithelial cells in the blood vessel wall to synthesize FSAP also remains a matter of debate [17,19,26,27,28,29]. By way of illustration, Nakazawa et al. mentioned unpublished data of Knoblauch et al. on the vessel wall synthesis of FSAP [26]. In turn, Daniel et al. did not find FSAP protein in normal murine arteries [27], but Sedding et al. detected the faint staining of FSAP antigen in the medial layer of murine arteries [17]. Both studies demonstrated the lack of FSAP mRNA in the arteries of mice [17,27], including the aorta, carotid artery [17,27], and femoral artery [17]. Sedding et al. concluded that the absence of FSAP mRNA in arteries indicated that FSAP protein was possibly incorporated from the circulation to the murine vasculature [17]. In agreement with studies on mice, Kannemeier et al. [28] and Parahuleva et al. [19,29] reported that human normal arteries do not express FSAP antigen. The sites of FSAP synthesis in tissues are shown in Figure 1a.

Human lymphocytes and dendritic cells have a very weak ability for FSAP mRNA expression [19]. More recently, isolated human platelets were shown to express FSAP mRNA and protein [29]. FSAP mRNA expression in platelets in vitro could be upregulated by their activators, including adenosine diphosphate (ADP) and thrombin receptor activating peptide (TRAP) [29]. These results demonstrated the possible direct relationship between platelet activation and FSAP, confirmed by the observation that acetylsalicylic acid (ASA), the inhibitor of the platelet activation and aggregation, reduced human platelet FSAP expression in vitro despite the presence of ADP and TRAP [29]. Thus, ASA therapy, e.g., in cardiovascular diseases, could potentially inhibit not only the platelet aggregation but also the platelet FSAP expression [29]; however, the clinical consequence of this axis is not fully understood.

The presence of FSAP mRNA in monocytes seems to be research model dependent [17,19,27,30,31,32]. FSAP mRNA was not seen in murine monocytes [17,27], whereas it was expressed by human monocytes [19,30,31,32]. As mentioned, Wygrecka et al. made an interesting observation that human alveolar macrophages were a minor source of FSAP mRNA, but mouse alveolar macrophages took up FSAP protein and metabolized it in vitro [21]. Contrastingly, in vitro-differentiated human macrophages had higher expression of FSAP mRNA than freshly isolated human monocytes in vitro [19]. Perhaps, the activation of the monocyte/macrophage lineage may lead to the changes in FSAP gene expression, but this speculation is yet to be confirmed. Furthermore, Parahuleva et al. detected FSAP protein in human monocytes and macrophages, simultaneously indicating the *de novo* synthesis of FSAP in these cells [19,30]. The ability of human monocytes [19,30,31,32] and macrophages [19,30,31] to express FSAP mRNA in vitro was stimulated by proinflammatory factors, including interleukin-6 (IL-6), interleukin-1α (IL-1α) [19], lipopolysaccharide (LPS) [19,30,31,32], and nicotine [30]. The addition of hormones to cell cultures, namely 17β-estradiol (E2) and progesterone (P4), increased FSAP mRNA expression in human macrophages [30,31]. In cultured human macrophages, one study also demonstrated the elevation of FSAP protein levels, which was induced by LPS, nicotine, 17β-estradiol, and P4 [30]. The hormone-driven FSAP upregulation in macrophages in vitro is in line with the higher plasma levels and activity of FSAP in women compared to men [33,34]. The higher FSAP mRNA expression in monocytes was also observed in women taking oral contraception (OC) [30,31]. The difference in plasma levels and activities between the sexes is described in detail later in this section of the manuscript.

The influence of the proinflammatory mediators may depend not only on the type of stimulating reactant but also on the type of stimulated cells and the condition of patients. In healthy human individuals, tumor necrosis factor α (TNF-α) did not change FSAP mRNA level in macrophages; however, the details of these experiments were not specified by the authors [19]. Contrastingly, the next paper from these authors described that in acute coronary syndrome (ACS) patients, TNF-α increased FSAP mRNA monocytic expression in vitro to a higher extent than in monocytes of healthy individuals [32]. Moreover, TNF-α, as well as IL-6, IL-8, and LPS but not IL-1β, induced the low baseline FSAP mRNA expression in cultured lung microvascular endothelial cells [21]. In cell culture of pulmonary endothelium, the induction of FSAP mRNA expression by LPS was in fact dependent on the LPS-mediated production of endogenous IL-8 [21], which is a well-known proinflammatory cytokine. Perhaps, this mechanism may be also responsible for the LPS-driven increased synthesis of the FSAP protein in pulmonary endothelial cells, which was described in another experiment conducted by Mambetsariev et al. [12].

In addition, the proinflammatory LPS-driven stimulation of low basal FSAP mRNA was also seen in vitro in human bronchial epithelial cells [21]. These in vitro data are in line with the pattern of FSAP pulmonary expression under pathological conditions [21,25]. As mentioned, it is controversial whether healthy animal and human lungs are able to produce FSAP [1,21,22,23,25]. However, the proinflammatory LPS-driven stimulation of FSAP mRNA was seen in vivo in murine pulmonary endothelium [12]. Moreover, during the administration of LPS to mice [12], when acute lung injury was induced in rats [25], and during acute respiratory distress syndrome (ARDS) in humans [21], FSAP was found in alveolar macrophages of bronchoalveolar lavage (BAL) fluid [21], endothelial cells of the lung [12,21,25] as well as bronchial [21] and alveolar [25] epithelial cells. Thus, some proinflammatory factors might possibly regulate the ability of pulmonary system cells to synthesize FSAP.

Apart from the mentioned lung endothelium, enhanced FSAP protein synthesis was observed in mouse brain microvascular endothelial cells (mBMECs) following hypoxia and reoxygenation [35]. However, FSAP synthesis was not seen in some other cell cultures [19,27]. Human umbilical vein endothelial cells (HUVECs) [19] as well as human [19,27] and murine [17] vascular smooth muscle cells (VSMC) were not able to exhibit FSAP mRNA expression at all, even after stimulation with inflammatory cytokines.

Similarly to monocytes/macrophages, the inflammatory stimulation via IL-6, IL-1, and LPS in vitro augmented FSAP mRNA expression in human trophoblast cells [24]. Finally, FSAP protein synthesis was observed in pathological cells, namely several types of human non-small cell lung cancer (NSCLC) [13].

Although FSAP synthesis in liver cells is commonly known, little is known about its regulation. FSAP mRNA expression in mouse hepatic cells was not altered by the following molecules: IL-1β, epidermal growth factor (EGF), platelet-derived growth factor-BB (PDGF-BB), basic fibroblast growth factor (bFGF), hepatocyte growth factor (HGF), connective tissue growth factor (CTGF), estrogen, and P4 [16].

Interestingly, the stimulation of mouse hepatic cells by transforming growth factor-β (TGF-β), a protein with anti-inflammatory properties, reduced FSAP mRNA expression and protein levels [16]. This TGF-β-mediated inhibitory effect involved its receptor (TGF-β-type 1 receptor, ALK-5) and SMAD2 signaling pathway, although FSAP promoter sequence has no SMAD binding sites [16]. Leiting et al. demonstrated that FSAP response to TGF-β was dependent on ATF3-binding site of the *HABP2* promoter region and accompanied by the reduced c-fos binding to the promoter [16]. The authors suggested that TGF-β increases ATF-3 and junB levels, which efficiently compete with c-jun/c-fos for the binding sites; binding of ATF-3 and junB heterodimer to the promoter region of *HABP2* would cause decreased FSAP mRNA [16]. On the other hand, the expression of HABP2 mRNA in mouse hepatic cells was elevated due to the treatment of cells with cyclic adenosine monophosphate (cAMP) pathway activators, including 8-(4-chlorophenylthio)adenosine 3′,5′-cyclic monophosphate sodium (8-CPT) and a plant-based substance forskolin (coleonol) [36]. It was suggested that the high levels of cAMP might influence the generation of FSAP mRNA via the activation of protein kinase A (PKA), which is a known cAMP activator-dependent kinase [36].

In summary, it may be argued that the inflammatory response can critically regulate FSAP. Sidelmann et al. even suggested that FSAP could possibly be an acute phase protein [37]. However, inflammation is not the only known regulatory mechanism of FSAP synthesis. Low molecular weight hyaluronic acid (LMWHA), which disrupts the endothelial barrier, increased the in vitro synthesis of FSAP in human pulmonary microvascular endothelium [12]. High molecular weight hyaluronic acid (HMWHA), which enhances vascular integrity, had exactly the opposite effect and reduced both FSAP mRNA and protein expression in vitro [12]. Therefore, mediators of vascular integrity appear to be important regulators of FSAP expression in vitro in endothelial cells.

### 1.2. FSAP Forms and Structure

Two forms of FSAP can be found in the animal and human plasma. Inactive FSAP (proFSAP), otherwise called single-chain FSAP (scFSAP) [9,10,11,17,18,19,21,23,25,28,38,39,40,41,42,43,44,45], circulates as a 60–78 kDa zymogen [1,2,11,21,38,39,40,42,46] consisting of 537 amino acids [1,11,38] following a 23 amino acid sequence of signal peptide [1]. Due to autocatalytic activation [11,17,38,45,47], the proenzyme splits between Arg290 and Ile291 [1,42] into the active form of 45-50 kDa [1,2,11,17,21,38,40] (290 amino acids of the heavy chain) [1,38] and 25-30 kDa [1,2,11,17,21,38,40] (247 amino acids of the light chain) [1,38] subunits linked by 18 [1,38] disulfide bonds [1,2,38]. This active heterodimer is called two-chain FSAP (tcFSAP) [2,9,10,11,17,18,21,23,25,28,38,39,40,41,42,43,44,45,48].

Structurally, FSAP is similar to hepatocyte growth factor activator (HGFA), though the implication of this similarity remains obscure [1,49]. Several regions of FSAP structure have been described, including the N-terminal region (NTR) [9,50], three EGF domains (EGF1, EGF2, EGF3) [1,2,9,12,46,47,50], the kringle domain [1,9,46,47,50], and the C-terminal serine protease domain [1,2,9,46,47,50]. FSAP structure is demonstrated on the Figure 1b. In the active form of the protein, the EGFs and kringle domains are located on the tcFSAP heavy chain, and the serine protease catalytic domain is located on the light chain of tcFSAP [1,2]. Although most of the original manuscripts have focused on the serine protease domain, which is related to its hemostatic functions, all FSAP domains are crucial for the proper functioning of the protein, especially in the interaction with various molecules in its activation process.

NTR was defined to extend from Phe1 to Pro53 with a high content of acidic (Glu8, Asp11, Asp13, Asp17, Asp20, Glu24, Asp25, Glu29, Glu30, Glu40, Asp43, Glu48, Asp49, and Asp52) and aromatic amino acids (Phe1, Trp14, Tyr19, Tyr21, Tyr23, Tyr26, Trp44, Tyr45, and Tyr46) [9]. NTR [9,10,50], or perhaps its subregion Glu40 to Asp52 [9], seems to be important during FSAP autoactivation [9,10,50], especially in cooperation with the EGF3 domain [9,50]. A significant role of EGF3 in the interactions with other molecules can be found in several studies [9,12,48,51,52], but the involvement of the EGF2 domain is less frequently mentioned [12,51]. The interactions of FSAP’s EGF3 with RNA [9,51] and heparin led to the binding of these molecules with FSAP [9,48,51,52] and activation of FSAP in vitro [9,51,52]. It was suggested that the three-dimensional (3D) structure of EGF3 is crucial for the binding between FSAP and its cofactors leading to the activation of this protein [51]. Nonetheless, tcFSAP generation in vitro might occur without cofactor participation [6,9,11,28,38,39,40,41,42,53]. According to Altincicek et al., the four amino acids within the EGF3 structure, Arg170, Arg171, Ser172, and Lys173 (the numbering includes 23 amino acids of the signal peptide), which belong to the exposed loop of this domain, interact with the negatively charged RNA through their positive charge [51]. The study of Yamamichi et al. [9] distinguished three additional cationic amino acids of EGF3 responsible for the heparin and RNA binding if compared to Altincicek et al. [51]. Thus, Yamamichi et al. described Arg144, His145, Lys146, Arg147, Arg148, Ser149, and Lys150 as components of the positively charged cluster of EGF3 [9]. It was suggested that the electrostatic interaction of acidic residues in NTR with basic amino acids in EFG3 of the scFSAP molecule could be the mechanism that protected the EFG3 domain from binding to another scFSAP molecule [9].

It seems that the least attention in the literature has been paid to the kringle domain, which was speculated to enable anti-angiogenic properties [8]. More specifically, it inhibits basic fibroblast growth factor (bFGF)-induced tube formation of HUVECs [8]. Stavenuiter et al. provided a more detailed analysis of the FSAP serine protease domain [14], which exhibited the highest homology to plasmin [54]. FSAP has a chymotrypsin folding, thus the serine protease domain is the model of dual antiparallel β-barrel [14]. The serine protease domain contains the catalytic triad, namely Ser195, His57, and Asp102 in chymotrypsin numbering [14]. The extension of the FSAP core has eight surface loops, which are considered to participate in the recognition of a substrate and regulate allosterically the catalytic activity [14]. The 220-loop together with the 180-loop (chymotrypsin numbering) may constitute the primary S1 specificity pocket, which is essential to identify the principle substrate residue [14]. The 220-loop is in proximity with the N-terminus insertion pocket, which stabilizes the S1 pocket after the proteolytic activation of the serine protease [14]. There are several possible sites in the serine protease domain, which could be responsible for the binding of the ions that propagate FSAP activity, namely Ca^2+^ and Na^+^ [14,47]. For Ca^2+^, it could be Asp64, Asp66, Glu70, and Glu71 [47] in the 70-loop (chymotrypsin numbering) [14,47]; for Na^+^, the probable binding site could be placed between the residues in the 220- and 180-loop [14,47]. Although much is known about the FSAP structure, these data are largely based on the analysis of the structure of the other chymotrypsins. Stavenuiter et al. mentioned that just the presence of the same structure in the protease domain of FSAP and other serine proteases does not mean that the significance of this structure is identical [14]. For this reason, further biochemical studies are needed to better understand the structure of FSAP.

The proper FSAP autoactivation is probably followed by some conformational changes [14]. Stavenuiter et al. proposed that the formation of the ion pairs between Ile16 (in chymotrypsin numbering), a newly formed N-terminus of the light chain, and Asp194 (in chymotrypsin numbering) of the serine protease domain is important for the successful FSAP activation [14].

### 1.3. FSAP Levels in Human Biological Samples

FSAP mean levels in human plasma are in the range from 5 to 12 µg/mL [10,11,33,38,44,46,55,56] or 80 to 200 nM [10,11,45]. More detailed quantification showed that FSAP levels, activity [33,34,57], and activity-to-levels ratio [34] are sex-dependent. The median levels are about 11.15 µg/mL (3.60–16.43 µg/mL) [33] or 97.4% [34] for women and 10.51 µg/mL (4.36–16.15 µg/mL) [33] or 87.5% [34] for men. The activity can be measured in plasma equivalent units per ml (PEU/mL) [33]. The median activity in females is 955 mPEU/mL (50 to 1453 mPEU/mL) [33] or 81.1% [34], and in males, it is 841 mPEU/mL (290 to 1326 mPEU/mL) [33] or 68.7% [34]. The ratio of the percentage FSAP activity to percentage FSAP levels was higher in healthy women in comparison with healthy men, i.e., 0.79 and 0.84 [34], respectively. Overall, women achieve higher values of FSAP measurements than men, which is consistent with the previously described relationships regarding the hormonal stimulation of FSAP synthesis by female sex hormones. Table 1 summarizes the mentioned data on FSAP levels and activity in healthy women and men.

FSAP measurements are characterized by inter-individual variability, e.g., due to genetic, lifestyle, and environmental factors. In women, FSAP measurements are partially endocrine-regulated [30,31,37,55]. The observed higher FSAP levels and activities in women [30,31,37,55] have been previously reported to be influenced by pregnancy [31], OC alone [30,31,37], OC usage combined with smoking [30], or hormone replacement therapy (HRT) [55]. Regardless of gender, the other potential regulators of the plasma FSAP levels were the following: higher body mass index (BMI) [57], hypertriglyceridemia, high fibrinogen levels [34,57], elevated levels of plasminogen activator inhibitor type 1 (PAI-1), thrombin activatable fibrinolysis inhibitor (TAFI), and TAFI activation peptide [57]. These connections further confirm the influence of inflammation and fibrinolysis on FSAP.

It was also verified whether FSAP could be found in specimens other than blood. The molecular weight of FSAP should not allow its glomerular filtration in the kidneys, and as expected, FSAP was not found in the urine of healthy humans and mice [58]. Similarly, FSAP was not detected in BAL fluids of healthy volunteers [21,39].

As shown in this paragraph, FSAP is a protease produced by different tissues. The predominant form circulating in the blood is inactive scFSAP, which can be activated to tcFSAP. The preservation of the appropriate structure of FSAP and the interaction of its domains, especially EGF3, with other molecules is paramount in protein activation. The detailed mechanisms of these processes are described in the next section of our review.

## 2. (Auto)activation and Activity Profile of FSAP

As mentioned previously, FSAP circulates in the plasma as a zymogen (scFSAP), in normal conditions [1,2,9,11,17,21,38,39,40,42,43,45,46,59]. If tcFSAP appears in the blood, it is evident that the activation of zymogen has occurred. FSAP activation leads to the generation of the active enzyme, and as a result, increased FSAP activity may be reported. In the literature, both studies on FSAP activation and FSAP activity can be found. FSAP activation experiments are designed to detect tcFSAP protein or tcFSAP-inhibitor complexes and test what molecules or conditions lead to the formation of tcFSAP from scFSAP [6,7,9,10,11,17,18,21,26,28,38,39,40,41,42,43,48,50,51,52,53,58,59,60,61,62,63,64,65,66,67,68,69,70,71]. FSAP activity tests exploit the ability of tcFSAP to perform various functions; they use tcFSAP to perform its functions under laboratory conditions and may verify the possibility to enhance or inhibit tcFSAP capabilities [2,3,9,11,18,25,28,39,42,46,48,52,53,54,60,67,70,72,73,74,75]. The following part of this review collects data on FSAP activation.

The process in which FSAP molecules bind and lead to tcFSAP generation was defined by Etscheid et al. as an intermolecular autoactivation [11]. It occurred in vitro between scFSAP and either scFSAP [38] or tcFSAP [9,11]. Yamamichi et al. demonstrated that in vitro FSAP molecules could bind together regardless of their active state [9]; however, the consequences of that phenomenon are not clear.

FSAP normally circulates in the plasma as a zymogen [1,2,9,11,17,21,38,39,40,42,43,45,46,59]. scFSAP isolated from plasma can auto-convert to tcFSAP [6,9,11,28,38,39,40,41,42,53]. Interestingly, the autoactivation of FSAP in vitro is faster in comparison with other chymotrypsin-type serine proteases [11]. If purified scFSAP was incubated together with FSAP-deficient plasma, no cleavage of scFSAP occurred [38]. Yamamichi et al. showed that the incubation of 80% (vol/vol) plasma with exogenous scFSAP in the presence or absence of exogenous tcFSAP did not lead to the generation of endogenous tcFSAP in plasma [9]. It was considered that this lack of effect was due to the FSAP inhibitors in plasma [9]. If the plasma was diluted to 10%, FSAP activation occurred, possibly via attenuated inhibition by the plasma serpins [9].

The question is if FSAP can be activated during the sample storage, thus distorting measured tcFSAP levels. Interestingly, there is a record of the storage influence on FSAP activation in the plasma samples [43]. Stephan et al. mentioned that blood clotting did not lead to the activation of plasma FSAP [43], but the details of this experiment were not shown. It was demonstrated that in healthy donors, no tcFSAP was detected even after 3 h of storage of plasma samples at 37 °C [43]. Therefore, the autoactivation of scFSAP did not start after the plasma collection from healthy subjects [43]. Pilot observations of these authors suggest that in septic patients, the level of tcFSAP was constant during sample storage at room temperature (within 3 h since blood collection), but the levels of tcFSAP increased if samples were incubated at 37 °C [43]. Thus, in specimens at room temperature, FSAP activation did not further progress despite the probable presence of circulating cell fragments [43], which might increase tcFSAP generation. These data indicate that, besides the patient’s condition, the storage temperature may affect FSAP activation. The observation that plasma or serum of septic patients contains tcFSAP started the search for the responsible mechanism of FSAP activation in septic blood.

scFSAP autoactivation generates tcFSAP, which, unlike scFSAP, has certain proteolytic activities. Normally, scFSAP is found in the blood, so the induction of scFSAP activation to tcFSAP may result in an increased FSAP activity. The evidence for the higher FSAP activity caused by other FSAP molecules was shown in vivo by Subramaniam et al. under pathological conditions [60]. In their experiment, the induction of carotid artery thrombosis in wild-type (WT) mice did not affect endogenous FSAP activity [60]. However, when human scFSAP was intravenously administered before the injury, an elevation of FSAP activity occurred [60], suggesting that injected exogenous scFSAP induced the generation of tcFSAP in vivo.

As a sign of the activation in vivo, tcFSAP was found in the plasma following liver injury and partial hepatectomy of mice [7]. Moreover, the activation of endogenous FSAP was seen in the plasma [21,43,59,61] or serum [62,63] of patients with ARDS [21], polytrauma patients [59], patients after transhiatal esophagectomy [43], melioidosis patients [61], subjects with sepsis [43,63] and septic shock [43], and during the low activity of systemic lupus erythematosus (SLE) [62]. The increase in tcFSAP in the patients mentioned above suggests a relationship between this protein and its activation with inflammation.

However, FSAP activation is not as obvious as it may seem because the level of tcFSAP generation does not always rise with the exacerbation of inflammation. The degree of FSAP activation in the blood remained constant [43], increased [43,61], or decreased [62] with the severity of the patient’s condition. In the study by Stephan et al., there were no FSAP activation differences between adults with septic shock versus severe sepsis [43], which differ in severity. Notwithstanding, the same manuscript demonstrated that the activation of FSAP in patients undergoing transhiatal esophagectomy was lower than in sepsis [43]; thus, significant differences in FSAP activation were seen between the low- and high-grade inflammation. As demonstrated by de Jong et al., from the hospital admission until convalescence, melioidosis patients exhibited a strong decline in tcFSAP generation towards the complete normalization [61]. Contrastingly, in sera of patients with the low SLE activity, FSAP activation was increased in the comparison with the high activity of the disease [62]. It shows that tcFSAP generation may be lower as the disease progresses, but the reason was unknown [62].

In terms of fatality in children with meningococcal sepsis, FSAP activation was higher in non-survivors than in survivors [43]. Nevertheless, other experiments did not find an association between FSAP activation and mortality in adults with severe sepsis and septic shock [43], nor in melioidosis [61]. de Jong et al. speculated that possibly, in severe melioidosis with advanced tissue damage, more tcFSAP was bound to dead cells and could not be detected when applying the assay used in the plasma [61]. The difference between the results of septic adults and septic children could be due to the greater homogeneity of the group of children who suffered from one specific type of sepsis, compared with the group of adults, which was heterogeneous and included patients with severe sepsis and septic shock from surgical and medical intensive care unit [43].

As mentioned in the previous subsection, FSAP was not found in the urine [58] or BAL [21,39] of healthy human subjects. The situation is different in pathological cases. The urine of nephrotic mice and patients contained the tcFSAP form [58]. Two independent studies by Wygrecka et al. detected tcFSAP in the BAL fluids [21,39] and the lung tissue [21] of ARDS patients. However, tcFSAP is not specific to all types of tissue damage, because it was not detected in murine plasma after kidney injury, though FSAP can be synthesized by this organ [7]. This absence of tcFSAP after kidney injury in murine plasma [7] relative to the mentioned presence of tcFSAP in the urine in the course of nephrotic syndrome [58] may also indicate differences between the biological samples of animals and humans.

Irrespective of the dynamics of changes in FSAP activation over time, the detection of endogenous tcFSAP indicates the activation of scFSAP in vivo. This occurrence seems to be closely related to inflammation, which motivates research on FSAP activation in various pathological states.

### 2.1. Enhancers and Inhibitors of FSAP Activation

FSAP can undergo autoactivation, but various factors in vitro and in vivo can accelerate or inhibit the FSAP autoactivation process. To the best of our knowledge, most of the studies on FSAP autoactivation involved in vitro conditions. The in vivo experiments were conducted much less often. Selected activators and inhibitors regulating FSAP activation are described below.

#### 2.1.1. Enhancers of scFSAP Activation

Over the years, several studies have shown that scFSAP activation is accelerated by heparin, a negatively charged glycosaminoglycan (GAG) [9,10,11,17,18,38,39,48,51,52,53,64]. The pioneering study by Etscheid et al. was the first to describe the enhancing effect of unfractionated heparin (UH) on the isolated FSAP autoactivation [11]. Various groups of researchers have performed mostly in vitro studies using purified or commercially available molecules [9,10,11,17,18,38,48,51,52,53,64]. It was demonstrated that UH [48,52], mast cell heparin, and low molecular weight heparin (LMWH) could bind to isolated FSAP [48]. These molecules propagate the autoactivation of isolated FSAP to varying degrees [48]. In contrast to UH [48] or native heparin [9,48], low molecular weight heparin (LMWH) had a minimal ability to act as the enhancer of isolated FSAP autoactivation. Moreover, LMWH specifically reversed the effect of spermidine, another propagator of tcFSAP generation, by inhibiting the spermidine-induced in vitro autoactivation of FSAP [9]. In turn, mast cell-derived heparin, which has a higher negative charge than UH, was more potent in terms of FSAP autoactivation increase in relation to UH [48]. The final conclusion of Muhl et al. was that the size, charge density, and conformational flexibility of polyanions appeared to be important for the interaction with isolated FSAP [48].

Contrary to the expectations based on the studies with purified systems, heparin failed to improve FSAP activation in human plasma [10,59,65]. Taking into consideration that sulfated glycosaminoglycans construct the extracellular matrix and are expressed by the endothelium [66], the ultimate role of heparin in FSAP autoactivation, especially in vivo, remains vague.

Importantly, FSAP activation could be influenced by the fibrinolysis regulator, uPA [38]. The generation of tcFSAP from purified scFSAP was accelerated by three forms of uPA, including uPA zymogen (single-chain uPA, scuPA), active uPA (two-chain uPA, tcuPA), and high molecular mass uPA (HMMuPA) [38]. Other studied isoforms, namely low molecular weight uPA, noncleavable and enzymatically inactive mutant scuPA-Gly158, and N-terminal fragment of high-molecular-mass urokinase (ATF) did not affect the activation of FSAP [38]. It indicated that the intact molecule and function of uPA were key to interacting with FSAP [38].

Choi-Miura et al. observed tcFSAP in the plasma of mice after hepatic failure and after partial hepatectomy [7]. To our knowledge, this study was the first to indicate a connection between FSAP activation and tissue injury [7]. The incubation of human plasma [43,67] or human serum [68] with apoptotic [43,67] or necrotic cells [40,43,68] caused the binding of dead cells to FSAP [43] followed by the activation of FSAP [40,43,67,68]. No significant activation of plasma FSAP by living human T lymphocyte cells (Jurkat cells) was seen [43]. However, the possibility of successful FSAP activation may depend on the cell culture, because Kannemeier et al. described that isolated scFSAP autoactivated both in the absence and presence of murine VSMC [28].

Some studies have focused on specific molecules that could emerge from dead cells and contribute to the generation of tcFSAP. In the purified system, highly cationic histones [10,63] and their subtypes promoted scFSAP autoactivation [10]. The strongest effect was demonstrated by histone 3 (H3), H2A, and H4 [10]. For example, the internal sequence of amino acid residues of H2A has been demonstrated to be cardinal for the acceleration of FSAP autoactivation [10]. Histones proved to be the powerful propagators of the activation of endogenous FSAP as was shown in human plasma [10,59,66,69,70] and sera [63] samples of healthy individuals. Animal studies support the activation of FSAP by histones [10]. The activation of FSAP in mice via the injection of histones [10] appears to be a breakthrough. This finding provided evidence of histone-promoted FSAP autoactivation occurring in vivo [10]. Based on both animal and human studies on sepsis, it was hypothesized the in vivo activation of FSAP could be due to the freed histones, but at that point, no unequivocal proof of this conception was demonstrated [10,63]. To confirm this hypothesis, further experiments and human studies are needed.

It is worth remembering that, besides free histone fractions, they can be complexed with DNA in various forms, namely, nucleosomes, chromatin, or neutrophil extracellular traps (NETs) [63,66,71]. It is not clear in which form histones are released from the damaged cell [63]. This can be critical in the context of tcFSAP formation, as not all histone forms lead to FSAP activation with comparable efficiency [59,63,66,71]. According to Semeraro et al., the DNA-histone complex promoted less effective plasma FSAP activation in vitro relative to the histones alone [66]. Marsman et al. focused on the FSAP activation by nucleosomes [63], which consist of DNA wrapped around a histone octamer. If the nucleosomes were predigested to release histones and then added to human serum, tcFSAP was detected [63]. However, after the incubation of the intact nucleosomes with the serum, the activation of FSAP was much weaker [63]. Presumably, the negatively charged DNA neutralized the positive charge of histones [63], impeding them to promote FSAP autoactivation.

Notwithstanding, the formation of tcFSAP in plasma correlated with the nucleosome levels in adults post-surgery (r = 0.55, *p* < 0.0001 and r = 0.64, *p* < 0.0001 depending on the measurement method of FSAP activation); patients with severe sepsis and septic shock (r = 0.43, *p* = 0.006 and r = 0.44, *p* = 0.004, depending on the measurement method of FSAP activation); children with meningococcal sepsis (r = 0.72, *p* < 0.0001 and r = 0.62, *p* < 0.0001 depending on the measurement method of FSAP activation) [43]; and polytrauma patients (r = 0.76, *p* < 0.001) [59]. FSAP activation in plasma correlated also with the nucleosome release in melioidosis subjects (r = 0.74, *p* < 0.0001) [61]. These reports [43,59,61] suggest a link between FSAP and nucleosomes, but they do not clarify the mechanism by which nucleosomes would activate FSAP.

Interestingly, the treatment of human plasma with chromatin, a more complex organization of nucleosomes, led to the activation of endogenous FSAP in vitro [59]. The histones, together with the chromatin DNA, are also components of NETs [71]. The formation of NETs is a type of cell death called NETosis, and it involves the release of nuclear factors [71]. Analogically to the nucleosomes [63], when purified FSAP or plasma was added to the neutrophils with induced NETosis, the binding between FSAP and NETs was seen; however, NETs formed during NETosis failed to alter FSAP activation in vitro [71]. Only if NETs were degraded by DNase and histones were freed, scFSAP conversion to tcFSAP in human plasma became significantly promoted [71]. As in nucleosomes [63], in NETs, DNA seemed to neutralize the histone functionality in the context of FSAP autoactivation [71]. Thus, in the case of more complex structures consisting of histones, histone accessibility seems to be essential to promote FSAP autoactivation.

Spermidine and spermine, which are positively charged polyamine compounds, can be released massively to the plasma during tissue injury or cellular death [9]. Spermidine increased intermolecular association between scFSAP and scFSAP, scFSAP and tcFSAP, as well as tcFSAP and tcFSAP [9]. Both spermidine and spermine enhanced FSAP autoactivation in vitro [9]. This feature is not equally presented by other tested polyamines, including putrescine, which exhibited little stimulatory function towards FSAP autoactivation [9]. Polyamines are elevated in the inflammatory tissues and malignant cells [9]. Thus, a rupture of such cells could possibly provide enough polyamines to facilitate FSAP autoactivation in vivo.

Nucleic acids, such as RNA, can also be released to the plasma as a result of cell damage [26]. Nakazawa et al. identified negatively charged extracellular RNA as the cell-derived cofactor for scFSAP autoactivation in vitro [26]. RNA bound both to scFSAP at multiple sites of the heavy and light chain and to tcFSAP within its heavy chain [26]. Important domains of FSAP enabling interaction with RNA are EGF2 and EGF3 [51]. FSAP at levels close to physiological values created RNA-FSAP complexes in vitro [26]; however, the minimal necessary fragment length of RNA to serve as a cofactor is 100 [26]–200 nucleotides [51]. FSAP autoactivation was accelerated by ribosomal RNA (rRNA) [26,51], mRNA [51], transfer RNA (tRNA), bacterial and viral RNA, as well as artificial RNA [26] in the purified system [26,51]. Notwithstanding, the study of Altincicek et al. showed that tRNA could augment the conversion of scFSAP to tcFSAP in vitro, but only under the condition of high levels of tRNA [51]. The first doubts about the significance of RNA in FSAP activation originated from the experiments reported by Zeerleder et al. [40] and Stephan et al. [43]. These studies showed the contribution of apoptotic and necrotic cells to generate tcFSAP in vitro [40,43]. The authors determined which cell-derived structure could be responsible for their observations [40,43]. RNA was excluded as an accelerator of FSAP activation because there was no difference in results between RNase-treated and untreated cells [40,43]. Two independent studies by Yamamichi et al. confirmed that the incubation of isolated scFSAP with RNA promoted FSAP autoactivation in the purified system [9,10], but there was no such effect existing in the human plasma [10].

Finally, there are contrasting data on DNA and FSAP activation. Semeraro et al. showed that DNA alone was not able to generate tcFSAP in the plasma [66]. Neither DNA homologue [26] nor DNA [71] was able to affect tcFSAP generation. DNA also did not form a complex with FSAP [26]. Conversely, Altincicek et al. noted that DNA had to be added at high levels to promote FSAP activation [51]. Apparently, the capability of nucleic acids to enhance FSAP autoactivation is controversial.

As FSAP autoactivation could be promoted by charged molecules, Sperling et al. conducted a study of endogenous FSAP activation on the extended planar material surfaces [50]. The authors demonstrated that cationic polyethylenimine (PEI) could induce FSAP autoactivation in the plasma and in whole blood samples [50]. Plasma FSAP activation was also seen on positively charged poly-L-lysine (PLL), but the effect was weaker in comparison to PEI [50]. FSAP activation was not present on the surface with negatively charged substrates such as glass and self-assembled monolayer with a carboxyl group (C(=O)OH), or with a neutral charge such as polytetrafluoroethylene (PTFE, teflon) [50]. Sperling et al. discussed that the higher activation of FSAP on PEI than on PLL can indicate how significant the charge density and chemical structure are for FSAP autoactivation [50]. It was concluded that cationic surfaces have comparable functionality to cationic macromolecules such as histones in terms of FSAP autoactivation [50]. As mentioned, there are negatively charged molecules that promote FSAP autoactivation in the purified system [9,10,11,17,18,26,38,48,51,52,53,64] though neither anionic molecules [10,59,65] nor extended flat anionic surfaces induced FSAP autoactivation in plasma [50]. The possible reasons for these observations were considered, namely an inadequate molecular conformation, the charge density of the anionic surface, or interfering plasma proteins [50]. Overall, this study is especially intriguing in terms of the safety of polycationic surfaces in medical devices and implants, as they could possibly lead to the generation of tcFSAP [50].

Factors accelerating FSAP autoactivation and their corresponding literature are summarized in Table 2.

Table 3 presents weak enhancers of FSAP activation or enhancers for which the data are contradictory.

**Figure 2:**
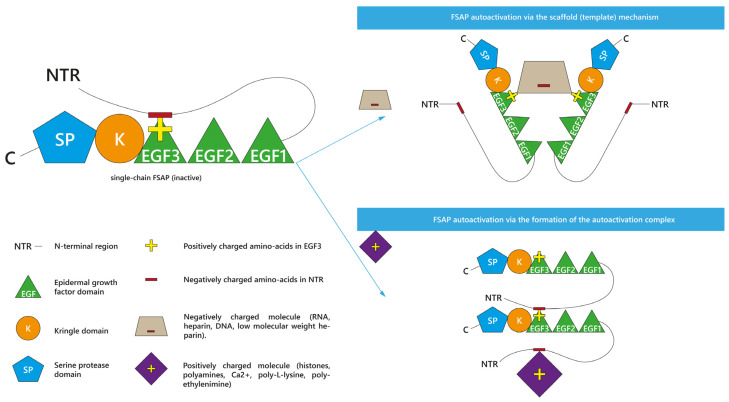
Two theoretical models of factor VII activating protease (FSAP) autoactivation. Negatively charged molecules promote FSAP autoactivation in vitro in the scaffold (template) mechanism or the interaction by binding. Positively charged molecules form the autoactivation complex together with FSAP molecules.

As presented, many various molecules are known as propagators of FSAP activation. Interestingly, two theoretical mechanisms of FSAP autoactivation have been proposed so far: the scaffold (template) mechanism [9,26,50,51] and the autoactivation complex model [9,10,50]. They are presented in Figure 2. The type of model depends on the positive or negative charge of the enhancer of FSAP autoactivation [9,50]. Both autoactivation mechanisms engage the NTR and EGF3 domains of FSAP [9,50]. Before the exploration of the details of these two models, it is important to understand the interactions between the domains of scFSAP. It was summarized that within the single inactive scFSAP molecule, the basic amino acids of the EGF3 domain (Arg144, His145, Lys146, Arg147, Arg148, Ser149, and Lys150) are guarded by the acidic (Glu8, Asp11, Asp13, Asp17, Asp20, Glu24, Asp25, Glu29, Glu30, Glu40, Asp43, Glu48, Asp49, and Asp52) and aromatic residues (Trp14, Tyr19, Tyr21, Tyr23, Tyr26, Trp44, Tyr45, and Tyr46) in the NTR [9]. This intramolecular interaction between positively charged EGF3 and negatively charged NTR protects scFSAP from binding to another scFSAP and thus prevents FSAP autoactivation [9,50]. However, the presence of some ionized molecules [9,50] or surfaces [50] can unblock EGF3 from NTR, making FSAP vulnerable to intermolecular autoactivation.

Irrespective of the results in plasma [10,59,65] and the study on anionic surfaces [50], negatively charged RNA [9,26,51] and heparin [9,51] promoted FSAP autoactivation in the purified system in the so-called scaffold (template) mechanism [9,26,51] or the interaction by binding [50]. In this model, an anionic molecule binds with the positively charged [50] EGF3 domains of different scFSAP molecules, forming a specific anionic bridge [9,26,50] between them. This anionic scaffold linking scFSAP molecules would facilitate their autoactivation [9,50]. As mentioned, LMWH had limited capability to accelerate FSAP autoactivation [9,48]. The possible reason could be surface of LMWH is too small to create a scaffold between two FSAP molecules [9].

The autoactivation complex is the second proposed mechanism [9,10,50]. It is based on the interaction with the cationic molecules, such as histones [10], and polyamines such as spermidine, spermine, or putrescine [9]. Because spermidine [9] and H3 [10] elevated the intermolecular binding of scFSAP, it was proposed that the positively charged molecules could form an autoactivation complex together with FSAP molecules [9]. More precisely, the presence of cationic molecules near scFSAP would lead to the release of the negatively charged NTR from EGF3 domain [9,50]. Thus, cationic molecules would allow for the intermolecular interaction of the released NTR of one FSAP molecule with the EGF3 of another FSAP molecule, leading to FSAP autoactivation [9,50]. This mechanism shows cationic molecules as promoters of the FSAP autoactivation complex [9,50].

It shall be highlighted that both the autoactivation complex and template mechanism are theoretical models and require further research.

#### 2.1.2. Inhibitors of scFSAP and Its Activation

Contrary to the propagation of scFSAP autoactivation, little is known about its inhibition. It remains obscure whether any natural inhibitor present in human blood is able to interact with and inhibit scFSAP as the data on this subject is fragmentary and, in some cases, contradictory. Wygrecka et al. even suggested that an effective interaction of the protease inhibitors with scFSAP is not expected due to the proteolytical inactivity of this form [39].

Serpins circulating in plasma at high levels are C1-inhibitor (C1-inh) and α-2-antiplasmin (AP) [10], but it is not known if they inhibit scFSAP activation. Shortly after FSAP discovery, Etscheid et al. indicated that AP and C1-inh caused a slower autoactivation of purified scFSAP [11]; however, to the best of our knowledge, it is the only study to provide such information. Furthermore, even the authors of this manuscript suspected that AP- and C1-inh-driven slowdown of FSAP activation was affected by the rapid inactivation of tcFSAP generated during the assay [11] instead of scFSAP. This assumption is in agreement with the common interpretation of the detected FSAP-inhibitor binding. Namely, the complexes between FSAP and AP [10,43,50,59,61,62,63,67,68,69,70,71] or C1-inh [43,67,70] are considered the markers of FSAP autoactivation in various in vitro and in vivo studies. Thus, although AP and C1-inh are not usually associated with scFSAP inhibition, their complexing with FSAP refers to the level of formed tcFSAP that is bound with the protease inhibitors.

An ambiguous observation concerning C1-inh, AP, and scFSAP can be found in the study by Kanse et al. [59]. In human plasma of healthy individuals, FSAP was co-immunoprecipitated not only with AP and C1-inh but also with many other inhibitors such as α2-macroglobulin, α1-trypsin inhibitor, and heparin cofactor 2 [59]. The significance of that result was not further examined, although the authors could not exclude that the mentioned inhibitors made complexes in vivo with scFSAP as the main FSAP form circulating in the plasma [59]. To verify this hypothesis, further studies should be conducted.

In the case of PAI-1, another member of the serpin family, it is also not clear whether it can inhibit the formation of tcFSAP from scFSAP [39]. Wygrecka et al. reported that PAI-1 did not bind or only exhibited limited binding with isolated scFSAP [39]. Moreover, this small level of FSAP-PAI-1 complexes could in fact be the result of the interaction between PAI-1 and tcFSAP, which autoactivated from scFSAP during the experiment [39].

As mentioned previously, LMWH is a weak enhancer of FSAP autoactivation [48], but it inhibits spermidine-induced FSAP autoactivation [9]. Table 4 summarizes FSAP activation inhibitors described in this subsection.

### 2.2. Enhancers and Inhibitors of FSAP Activity

FSAP is a serine protease that circulates in the blood as a proteolytically inactive zymogen. To perform protease activities efficiently, the zymogen must become active; in other words, scFSAP must progress into tcFSAP [10,23,39,40,41,42,52,59,64]. Therefore, the activation leads to an increase in FSAP activity in normal conditions. Studies on FSAP activation analyze the formation of tcFSAP from scFSAP, while activity measurements verify various functions of the already generated tcFSAP. Activity experiments are possible by the direct use of purified tcFSAP or an induced activation of endogenous scFSAP before the measurement. This subsection is focused on the numerous enhancers and inhibitors of FSAP activity [2,3,9,11,18,25,28,39,42,46,48,52,53,54,67,70,72,73,74,75]. The majority of them were reported in in vitro studies. It is worth mentioning that this subsection refers to the unspecified activity of FSAP. FSAP performs many functions, so the term “activity” is general and not specific. Importantly, different studies used various FSAP functions to evaluate the efficiency of potential activators and inhibitors of its activity.

#### 2.2.1. Enhancers of tcFSAP Activity

Heparin was studied as the potential enhancer of tcFSAP activity, but the conclusions are not always consistent [3,9,18,25,28,42,46,48,52,72,73,74,75]. In many cases, the addition of heparin [3,18,25,28,42,46,48,52,73,74,75] intensified the activity of tcFSAP in vitro. Without heparin, FSAP was even unable to perform various activities in cultured human pulmonary fibroblasts (HPF) [25] and primary hepatic stellate cells (HSC) [18].

However, the effect of heparin can differ under the specific conditions of the experiment, such as the type of cell culture [75]. Heparin presence was necessary for FSAP activity in human lung carcinoma epithelial (A549) cells; however, in human embryonic kidney (HEK293T) cells, the influence of heparin on FSAP activity was insignificant [75]. The ambiguous reports about heparin may depend on the time of the measurement. Heparin increased the inhibitory activity of FSAP on DNA synthesis in mouse [48,73] and human VSMC [28,73]. However, if the number of human VSMC was assessed in long term, i.e., after 5 days, heparin failed to propagate FSAP activity [28].

It remains controversial whether LMWH can enhance FSAP activities. LMWH promoted FSAP activity in mouse VSMC [48] but failed to do so in human VSMC [28]. Similarly, with regard to FSAP autoactivation, even if LMWH promoted FSAP activity in the study by Muhl et al., it did so to a lesser extent than UH [48].

In summary, it cannot be unequivocally answered whether heparin is an enhancer of FSAP activity or not. The influence of heparin and LMWH on FSAP activity appears even more complicated if one would consider that these polyanions have been linked to the inhibition of FSAP activity [54] or cooperation with tcFSAP inhibitors in some experiments [11,46,48,52]. This inhibitory effect of heparin and LMWH is described further in the next subsection.

In the case of the nucleic acids, RNA enhanced FSAP activity [73]; however, it also served as the cofactor of the inhibitor-driven reduction of tcFSAP activity [39]. In comparison, the influence of DNA on FSAP activities was reported as weakly promoting or even ineffective [73]. Although histones were shown to make a significant impact on FSAP autoactivation, their effect on FSAP activity was not seen [10]. Similar findings concern other cationic molecules, i.e., polyamines [9]. While they were potent to accelerate FSAP autoactivation in vitro, the polyamine-driven stimulation of the FSAP activity was only minimal [9]. It shows that inducers of FSAP autoactivation and enhancers of FSAP activity can be divergent.

The mentioned enhancers of FSAP activity are presented in Table 5.

#### 2.2.2. Inhibitors of tcFSAP and Its Activity

By 1999, it was already clear that human plasma had some inhibitory capability towards FSAP activity, as indicated after incubation of different plasma dilutions with isolated tcFSAP [2]. In the experiment evaluating variety of serpins, AP and C1-inh were distinguished as the inhibitors of tcFSAP activity [2]. As mentioned previously, FSAP complexes with AP and C1-inh are used as markers of tcFSAP level generated by autoactivation, but the purpose of this complexing is to limit the activity of tcFSAP. Therefore, tcFSAP-inhibitor binding is connected with the reduced tcFSAP activity. Hunfeld et al. showed that AP was a more effective inhibitor of FSAP activity than C1-inh in the purified system [2]. However, shortly after that, Choi-Miura et al. isolated and determined C1-inh as the major, natural inhibitor of tcFSAP in plasma [6].

tcFSAP forms in vitro complexes with AP [10,39,43,50,59,61,62,63,67,68,69,70,71] and C1-inh [6,39,43,67,70], which is in line with AP- [2,11,39,46,67] and C1-inh-driven [2,39,46,67,70] reduction of FSAP activity in vitro. It was shown that the complex formation of tcFSAP with AP [10,43,50,59,61,62,63,69] or C1-inh [43] can be induced via the incubation in vitro of the animal or human plasma [10,43,50,59,61,69,70], serum [62,63], or whole blood [50] with a propagator of FSAP activation, such as apoptotic cells [43,61,62], histones [10,59,63,69,70], PEI [50], or chromatin [59]. In healthy humans’ plasma treated with apoptotic cells, the median of complexes’ levels was 0.5 AU/mL (0.5 AU/mL ± 0.03 for FSAP-AP, and 0.5 AU/mL ± 0.04 for FSAP-C1-inh) [43]. De Jeong et al. reported a mean of 0.03 AU/mL for FSAP-AP complexes in healthy controls [61].

The inactive, circulating form in the normal blood is scFSAP [9,10,11,17,18,19,21,23,25,38,39,40,41,42,43,44,45], which, in contrast to tcFSAP, is unlikely to bind with protease inhibitors [6,10,39,43,50,59,61,62,63,67,68,69,70,71]. Therefore, one can expect none or trace amounts of tcFSAP-serpin complexes in the plasma under normal conditions. In line with this assumption, in the healthy human plasma untreated with any FSAP autoactivation enhancer, Stephan et al. did not detect FSAP-AP and FSAP-C1-inh complexes [43]. Thus, if endogenous scFSAP in plasma did not proceed into tcFSAP, no serpin binding is observed.

The intravenous application of human tcFSAP to mice led to the formation of complexes between human tcFSAP and plasma inhibitors in vivo [17]. Moreover, histone-injected mice had increased formation of tcFSAP-AP complexes in their plasma samples in relation to unstimulated mice, whereas the complexing of FSAP with C1-inh seemed insignificant [10]. The accumulation of FSAP-AP complexes in plasma was also detected in the course of the in vivo murine model of LPS-induced sepsis [10]. Interestingly, it seems that there are negligible levels of FSAP-C1-inh complexes in mice after histone injection [10]. Both C1-inh and AP are abundant plasma proteins; however, for an unknown reason, FSAP was predominantly bound in vivo by AP under these experimental conditions [10]. Yamamichi et al. suspected some histone-dependent-favoring mechanism of FSAP neutralization by AP [10].

There are several data concerning the complex formation in vivo between tcFSAP and serpin inhibitors in various disorders among humans. If compared with healthy controls, increased complexes of FSAP-AP were detected in the plasma of melioidosis patients [61], polytrauma patients immediately after injury [59], post-surgery patients, and patients with severe sepsis and septic shock [43]. Similarly, FSAP-AP complexes were found in the sera of subjects with meningococcal sepsis [63] and SLE [62], while significantly higher levels of FSAP-C1-inh complexes were seen in the plasma of patients after surgery, subjects with severe sepsis and septic shock, and meningococcal sepsis patients [43]. Apparently, C1-inh and AP are not universal inhibitors of FSAP in all types of biological material [39]. In BAL fluid of ARDS patients, FSAP was not co-immunoprecipitated with C1-inh and AP [39]. Wygrecka et al. considered that such complexes possibly do not occur in BAL or could not be detected due to the methodological procedure [39]. Perhaps, it would be useful to measure tcFSAP-inhibitor complexes in the blood of ARDS patients.

According to animal and human studies, tcFSAP can form in vivo complexes both with AP and C1-inh during certain diseases and pathological conditions. However, the exact mechanisms are unknown. It remains obscure whether the absence of FSAP-C1-inh complexes after histone injection in mice is due to the histone-dependent favoring of AP as an FSAP inhibitor or a mouse-specific observation. The possibility of different pathways leading to scFSAP activation and subsequent inhibitory responses to tcFSAP should be elucidated in the future.

tcFSAP inhibition seems to be related to hemostasis and more specifically to the inhibition of fibrinolysis. Next to AP and C1-inh, PAI-1 is another serpin that formed a 1:1 stoichiometric complex with tcFSAP, inhibiting its activity in the purified system [39,48,52] and in cell culture [39]. The pilot data reported by Muhl et al. mentioned that FSAP-PAI-1 complex could bind to low-density lipoprotein receptor-related protein (LRP) and become internalized by cells [72]; however, any further detailed report is not available to the best of our knowledge. For reference, Römisch et al. demonstrated that PAI-2 and PAI-3 did not inhibit isolated tcFSAP [46].

Surprisingly, the inhibition of tcFSAP by PAI-1 was much more marked, if RNA, and to a lesser extent, vitronectin, or DNA were added to the measured system [39]. It is intriguing because, as mentioned before, RNA was the propagator of scFSAP autoactivation [9,10,26,51] and tcFSAP activities [73] according to some studies. The stimulatory effect on FSAP inhibition could probably be attributed to the RNA- and vitronectin-driven protection of PAI-1 from degradation [39].

In the in vivo model of histone-injected mice, Yamamichi et al. detected an insignificant level of FSAP-PAI-1 complexes, which could be explained by the relatively low levels of PAI-1 in plasma [10]. Notwithstanding, among the healthy human population, PAI-1 antigen positively correlated with FSAP antigen levels (r = 0.16, *p* < 0.001) and FSAP activity (r = 0.11, *p* = 0.006) in plasma [57], but the significance of this phenomenon is unknown. In septic patients, PAI-1 and C3a, as the predictive parameters for sepsis outcome and inflammatory markers, correlated with FSAP-inhibitor complexes (r > 0.33, *p* < 0.05), showing these complexes to increase with the stage of inflammation [43]. In healthy subjects, FSAP barely occurs in their BAL fluids [21,39], but the complex of PAI-1 and FSAP was precipitated from the BAL fluids of the patients with ARDS [39]. Thus, the formation of FSAP-PAI-1 complexes in vitro was confirmed in the in vivo study. As RNA promoted tcFSAP inhibition by PAI-1 in vitro and BAL of ARDS patients contained a significant increase in RNA levels in relation to healthy subjects, the authors discussed the possible relationship between RNA, tcFSAP, PAI-1, and ARDS in vivo [39]. The disruption of cells in ARDS may release nucleic acids; then, RNA may stabilize and prolong the inhibitory activity of PAI-1 as well as promote FSAP-PAI-1 binding [39]. The clinical implications of this FSAP inhibition by PAI-1 remain not fully understood; however, PAI-1 may regulate FSAP functions related to hemostasis and cell biology [39].

The inhibition of FSAP has been demonstrated by another serine protease inhibitor present in the endothelium, i.e., protease nexin-1 (PN-1) [72]. Similar to other described serpins in this paper, PN-1 bound to isolated tcFSAP [72] and prohibited in vitro some enzymatic FSAP activities [72,73]. Muhl et al. provided data showing that the inhibition of FSAP by PN-1 might involve cell scavenger receptors such as LRP [72]. The FSAP-PN-1 complex, but not FSAP alone, is specifically and strongly bound to LRP [72]. Subsequently, the complex was internalized by mouse embryo fibroblasts (MEFs) and mouse VSMC in vitro via this scavenger receptor and directed to lysosomes [72]. This intracellular regulation of FSAP may be a mechanism of clinical relevance that would explain high, local levels of FSAP inside the cells under pathological conditions.

As mentioned in the previous subsection, heparin, and LMWH do not seem to be universal propagators of FSAP activity—especially if a polyanion and FSAP exhibit an opposite influence on a substrate. A study by Roedel et al. reported reduced FSAP activity in the presence of heparin [54]. While purified FSAP promoted the bone morphogenetic protein-2 (BMP-2) function in vitro, heparin alone decreased the functionality of BMP-2 [54]. In this experiment, heparin did not enhance FSAP activity towards BMP-2, as the final result of the FSAP-heparin co-incubation was the diminution of BMP-2 function [54]. This observation might not imply a direct inhibition of tcFSAP by heparin; however, if heparin regulates the functionality both of FSAP and the FSAP’s substrate, such as BMP-2, the heparin-driven promotion of tcFSAP activity may not be seen.

Furthermore, heparin promotes the interaction between tcFSAP and its inhibitors. The inhibition of FSAP activity by AP [11] and antithrombin (AT) [11,48] was increased by heparin. This is not a general relationship because heparin did not alter the inhibition of FSAP activity by PN-1 [72]. AT represents an interesting phenomenon, as this inhibitor alone does not form a complex with tcFSAP [39], nor does it affect scFSAP autoactivation [11] or tcFSAP activity [2,39,46,48]. However, under the presence of a heparin-like negative charge density [48], AT is potent to inhibit tcFSAP in vitro [11,46,48]. Intriguingly, the preincubation of AT with LMWH led to the opposite effect, i.e., the increased in vitro activity of tcFSAP [48]. It shows a certain selectivity of the specific polyanions towards the inhibitors of tcFSAP [48].

Some studies have associated heparin and LMWH with another FSAP inhibitor, PAI-1 [48,52]. Although no such effect was reported by Wygrecka et al. [39], Muhl et al. showed that UH [48,52] and LMWH [48] increased PAI-1-driven inhibition of tcFSAP activity in vitro. This heparin-stimulated inhibitory action of PAI-1 is dependent on the EGF-3 domain of FSAP [52], but the exact implication of this observation remains unknown. The EGF-3 domain is important for the interaction with anionic molecules like heparin and is essential for FSAP autoactivation; thus, there is a possibility that EGF-3 directly participated in the heparin-boosted inhibition of FSAP by PAI-1.

The general reasons for the dualistic influence of heparin on FSAP activity are puzzling. It was concluded that polyanions promote the inhibition of FSAP activity because they change the conformation of the serpins and support their binding with FSAP [48]. Therefore, polyanions can enhance the autoactivation and activity of isolated FSAP, but they can also facilitate the interaction of tcFSAP with its inhibitors.

Tissue factor pathway inhibitor (TFPI) is a Kunitz-type inhibitor, impeding blood coagulation via the inactivation of activated factor VII (FVIIa) and X (FXa) [41,60,67]. Although TFPI was initially not shown to alter the activity of purified FSAP [46], that possibility was further re-examined [67]. Surprisingly, TFPI inhibited the activity of purified FSAP in vitro 300 times more efficiently than AP and C1-inh [67]. In the environment of apoptotic cells, TFPI reduces the nucleosome release activity of both purified and plasma FSAP with equal efficiency [67]. As the more effective inhibitor than AP and C1-inh, TFPI restrained the formation of complexes between tcFSAP and C1-inh or AP [67]. The causation of the difference in efficiency between TFPI, C1-inh, and AP was not proven, but distinct mechanisms of action between serpins and Kunitz-type inhibitors can be suspected [67]. Notably, Stephan et al. highlighted the usage of supraphysiological levels of plasma TFPI throughout their study [67], which might influence the results. Notwithstanding, such high TFPI levels occur in vivo locally on the cellular surfaces [67], suggesting a possibility of the local interaction between FSAP and TFPI.

TFPI consists of 3 Kunitz-type domains [41,60,67]. Stephan et al. showed that the C-terminal region of TFPI, known as heparin or cell surface binding site, was the key for direct binding to FSAP [67]. That binding probably enabled further FSAP inhibition by TFPI [67]. TFPI domains were key to FSAP inhibition. The most important was Kunitz-type domain 2 (K2) followed by the C-terminal region and to a lesser extent, Kunitz-type domain 3 (K3) [67]. The significance of Kunitz-type domain 1 (K1) was described as partial or none depending on the measurement method [67].

Kanse et al. showed that TFPI is a substrate that is cleaved and inactivated by FSAP [41]. Taking this report into account [41], the mechanism of TFPI-driven inhibition of FSAP could have been in fact the FSAP-mediated proteolysis of TFPI. The potential cleavage of TFPI by FSAP could have interrupted the reaction between FSAP and its target substrate [67]. In such cases, the measured decrease of FSAP activity could have been the result of the competitive mechanism between two FSAP substrates, namely TFPI and the target substrate of the assay. However, Stephan et al. excluded the possibility that TFPI could have been proteolyzed by FSAP [67]. The authors revealed that when FSAP was co-incubated with TFPI, FSAP activity did not increase over time, suggesting a constant presence of TFPI [67]. Thus, Stephan et al. concluded that TFPI was an inhibitor of FSAP in their experiment [67].

Described inhibitors of tcFSAP and tcFSAP activity are listed in Table 6.

Table 7 includes tcFSAP inhibitors for which the data are contradictory.

As shown in this section, scFSAP is the predominant form in plasma under normal conditions. scFSAP progresses into the active form called tcFSAP during intermolecular autoactivation. The scaffold mechanism and autoactivation complex are two theoretical models that describe the suspected mechanism of FSAP autoactivation. The measurement of tcFSAP generation, i.e., autoactivation is analyzed in many studies. tcFSAP as the active enzyme performs various activities that are measured during activity assays. Both scFSAP autoactivation and tcFSAP activity are controlled by many enhancers and inhibitors, but the majority of them were reported in in vitro studies. Moreover, for some enhancers and inhibitors, experimental data are conflicting. Thus, the significant implications of these experiments in in vivo settings are still unknown.

## 3. Conclusions

The review of the literature clearly demonstrates that FSAP is a protein with unique properties, whose role in hemostasis has yet to be determined. Part I of our manuscript describes the structure, activation, and activity of FSAP. The second part focuses on the importance of FSAP for coagulation and fibrinolysis. Part III is devoted to FSAP-driven modulation of the inflammatory state, as well as the relationship between the presence of FSAP polymorphisms and the role of this protein in the physiopathology of human diseases, with a particular emphasis on cardiovascular events.

## Figures and Tables

**Table 1:** Factor VII activating protease (FSAP) levels, activity, and the ratio of percentage FSAP activity to percentage FSAP levels in human plasma.

Measurement	In Women	In Men	*p*-Value (Women vs. Men) *	References
FSAP levels	11.15 µg/mL (3.60–16.43 µg/mL)	10.51 µg/mL (4.36–16.15 µg/mL)	0.07	[33]
	97.4%	87.5%	<0.001	[34]
FSAP activity	955 mPEU/mL (50–1453 mPEU/mL)	841 mPEU/mL (290–1326 mPEU/mL)	0.0005	[33]
	81.1%	68.7%	<0.001	[34]
The ratio of percentage FSAP activity to percentage FSAP levels	0.79	0.84	<0.001	[34]

* FSAP levels (µg/mL) and activity (mPEU/mL) were compared between men and women with Wilcoxon’s rank sum test (two-sided *p* values) [33]; FSAP levels (%), activity (%), and the ratio of percentage FSAP activity to percentage FSAP levels were compared between the groups with Dunn test [34].

**Table 2:** Enhancers of single-chain factor VII activating protease (scFSAP) activation.

Name	Studies In Vitro Describing the Influence
Single-chain urokinase plasminogen activator (scuPA)	[38]
Two-chain urokinase plasminogen activator (tcuPA)	[38]
High molecular mass urokinase plasminogen activator (HMMuPA)	[38]
Histones	[10] ^1^, [59,63,66,69,70,71]
Nucleosomes (predigested)	[63]
Chromatin	[59]
Polyamines (spermidine, spermine, putrescine)	[9]
Neutrophil extracellular traps (NETs) (disintegrated)	[71]
Apoptotic and necrotic cells	[40,43,67,68]
Poly-L-lysine (PLL)	[11,50]
Polyethylenimine (PEI)	[50]

^1^ The effect was described in vitro and in vivo.

**Table 3:** Weak enhancers of single-chain factor VII activating protease (scFSAP) activation and enhancers for which data are contradictory.

Name	Studies In Vitro Describing the Influence	Contradictory Data
RNA	[9,10,26,51]	RNA was excluded as the cell-derived structure promoting FSAP activation [40,43]. RNA failed to promote FSAP activation in human plasma [10].
DNA	Effect at high levels of DNA [51]	Neither DNA homologue [26] nor DNA [66,71] was able to affect FSAP activation in vitro.
Heparin	[9,10,11,17,18,38,48,51,52,53,64]	Heparin failed to promote FSAP activation in human plasma [10,59,65].
Low molecular weight heparin (LMWH)	Weak effect [48].	LMWH inhibited spermidine-induced FSAP autoactivation in vitro [9].

**Table 4:** Single-chain factor VII activating protease (scFSAP) activation inhibitors for which data are contradictory.

Name	Studies In Vitro Describing the Influence	Contradictory Data
α-2-antiplasmin (AP)	[11]	FSAP-AP complexes are considered as the marker of completed FSAP activation [10,43,50,59,61,62,63,67,68,69,70,71].
C1-esteraze inhibitor (C1-inh)	[11]	FSAP-C1-inh complexes are considered as the marker of completed FSAP activation [43,67,70].
Plasminogen activator inhibitor type 1 (PAI-1)	Limited scFSAP-PAI-1 binding [39].	Another experiment of the same authors indicated that scFSAP did not bind with PAI-1.
Low molecular weight heparin (LMWH)	LMWH inhibited spermidine-induced FSAP autoactivation [9].	LMWH weakly promotes FSAP activation [48].

**Table 5:** Two-chain factor VII activating protease (tcFSAP) activity enhancers for which data are contradictory.

Name	Studies In Vitro Describing the Influence	Contradictory Data
RNA	[73]	RNA increased inhibitor-driven reduction of tcFSAP activity [39].
DNA	Weak effect [73].	DNA did not alter FSAP activity effectively [73].
Polyamines	Minimal effect [9].	nd ^1^
Heparin	[3,18,25,28,42,46,48,52,73,74,75]	Heparin did not alter significantly FSAP activity [9,28,72,75] or FSAP activity was reduced in heparin presence [54]. Heparin increased inhibitor-driven reduction of tcFSAP activity [11,46,48,52].
Low molecular weight heparin (LMWH)	[48]	LMWH did not alter significantly FSAP activity [28]. LMWH increased inhibitor-driven reduction of tcFSAP activity [48].

^1^ nd = Not determined.

**Table 6:** Inhibitors of two-chain factor VII activating protease (tcFSAP) and its activity.

Name	Studies In Vitro Describing the Influence
α-2-antiplasmin (AP)	[2,10] ^1^, [11,39], [43] ^1^, [46,50], [59] ^1^, [61,62,63] ^1^, [67,68,69,70,71]
Plasminogen activator inhibitor-1 (PAI-1)	[39] ^1^, [48,52,72]
Protease nexin-1 (PN-1)	[72,73]
Antithrombin (AT) + heparin	[11,46,48]

^1^ The relationship was described in vitro and in vivo.

**Table 7:** Inhibitors of two-chain factor VII activating protease (tcFSAP) and its activity for which data are contradictory.

Name	Studies In Vitro Describing the Influence	Contradictory Data
Heparin	[11,46,48,52]	Heparin promoted [3,18,25,28,42,46,48,52,73,74,75] or did not alter FSAP activity [9,28,72,75]. Heparin did not promote PAI-1-driven inhibition of FSAP [39].
Low molecular weight heparin (LMWH)	[48]	LMWH did not alter significantly [28] or increased tcFSAP activity [48].
C1-esterase inhibitor (C1-inh)	[2,6,39,43] ^1^, [46,67,70]	Murine plasma levels of tcFSAP-C1-inh complexes were insignificant after histone injection [10].
Tissue factor pathway inhibitor (TFPI)	[67]	TFPI failed to inhibit tcFSAP activity [46].

^1^ The relationship was described in vitro and in vivo.

## Data Availability

Not applicable.
